# Telemental Health in Low- and Middle-Income Countries: A Systematic Review

**DOI:** 10.1155/2018/9602821

**Published:** 2018-11-01

**Authors:** Jeremiah W. Acharibasam, Rolf Wynn

**Affiliations:** ^1^Department of Mental Health, Navrongo War Memorial Hospital, Navrongo, Kassena-Nankana District, Ghana; ^2^Department of Clinical Medicine, Faculty of Health Sciences, The Arctic University of Norway (UiT), N-9037 Tromsø, Norway; ^3^Division of Mental Health and Addictions, University Hospital of North Norway, N-9038 Tromsø, Norway

## Abstract

**Introduction:**

The rising incidence of mental illness and its impact on individuals, families, and societies is becoming a major public health concern, especially in resource-constrained countries. Consequently, there is an increasing demand for mental health services in many middle- and low-income countries (LMIC). Challenges such as inequality in access, lack of staff and hospital beds, and underfunding, often present in the LMIC, might in part be addressed by telemental health services. However, little is known about telemental health in the LMIC.

**Methods:**

A systematic review was performed, drawing on several electronic databases, including PubMed, PsycINFO, Web of Science, Springer Link, and Google Scholar. Original English language studies on the practice of telemental health in LMIC, involving patients and published between 1 January 2000 and 16 February 2017, were included.

**Results:**

Nineteen studies met the inclusion criteria. Most of the articles were recent, which may reflect an increasing focus on telemental health in the LMIC. Eight of these studies were from Asia. Eight of the studies were interventional/randomized controlled trials, and 11 examined general mental health issues. Videoconferencing was the most frequently (6) studied telemental modality. Other modalities studied were online decision support systems (3), text messaging and bibliotherapy (1), e-chatting combined with videoconferencing (1), online therapy (2), e-counseling (1), store-and-forward technology (1), telephone follow-up (1), online discussion groups (1), audiovisual therapy and bibliotherapy (1), and computerized occupational therapy (1). Although many of the studies showed that telemental services had positive outcomes, some studies reported no postintervention improvements.

**Conclusion:**

The review shows a rising trend in telemental activity in the LMIC. There is a greater need for telemental health in the LMIC, but more research is needed on empirical and theoretical aspects of telemental activity in the LMIC and on direct comparisons between telemental activity in the LMIC and the non-LMIC.

## 1. Introduction

The demand for adequate mental healthcare is increasing in many low- and middle-income countries (LMIC) [1–3]. The LMIC are countries that according to the World Bank's classification have a per capita gross national income of less than USD 12,056 [4]. The LMIC are faced with several barriers that limit access to quality healthcare services including long distances between healthcare centers, underfunding, lack of highly qualified clinicians and hospital beds, low mental health awareness, and high levels of illiteracy in parts of the population [1–3]. Telemedicine and e-health might help alleviate some challenges facing the healthcare services in the LMIC [5, 6] and might be particularly useful in mental healthcare [1–3, 5, 6].

Telemental health traditionally involves the provision of mental healthcare across a distance using communication technologies [7, 8] and has been practiced for more than 50 years [9]. Videoconferencing is by far the most common and the most researched type of telemental health, but other types, such as text messaging or the use of mobile apps, are on the rise [10–12]. Studies conducted mainly in developed countries have found that the effectiveness of telemental health (i.e., videoconferencing) is comparable to traditional face-to-face services [13–16]; this applies to the reliability of assessments, treatment outcomes [17], and satisfaction [18]. Some patient groups may even prefer telemental health services above traditional services [9, 19]. Telemental health services are also cost-effective [17, 20]. However, some reviewers have emphasized the lack of high quality evaluations of telemental health services [21, 22].

Telemental health offers the LMIC a unique opportunity that may improve access to quality mental health services including enabling remote care delivery, expanding access to qualified mental health personnel, improving clinical supervision and training, promoting cost and time efficiency of care, and enhancing clinician daily workflows and clinical routines [1–3, 23–26]. However, little is known about the scope of use or the outcomes of telemental health in the LMIC. In order to address this lack of overview, we performed the present review.

## 2. Methods

This review followed a systematic literature review approach guided by the Preferred Reporting Items for Systematic Reviews and Meta-Analyses (PRISMA) [27].

### 2.1. Inclusion and Exclusion Criteria

We included articles published in the English language that had a main focus specifically on telemental health, that were conducted in low- and middle-income country settings, that involved human subjects, and that were published as primary studies between 1 January 2000 and 16 February 2017. In order to capture as many relevant articles as possible, we adopted a broad definition of telemental health technologies to include also mental health-related online decision support systems, text messaging (SMS), online therapy, e-chatting, e-counseling, online support forums, and relevant computer programs/apps.

We excluded articles that were published in languages other than English, that had a main focus outside of telemental health, that were conducted in non-LMIC settings, that did not involve human subjects, that were review studies, commentaries, letters, and editorials, that were duplicates of included articles, and that were not published between 1 January 2000 and 16 February 2017.

### 2.2. Search Strategy

A comprehensive literature search was conducted on several databases including Web of Science, Google Scholar, Psych INFO (Ovid), PubMed, and Springer Link. Furthermore, a rigorous hand search for additional relevant literature was carried out to help identify grey literature including project evaluation reports. The same key terms were used in searches conducted on the various databases including telepsychiatry, e-mental health, telemental health, behavioural telemedicine, virtual rehabilitation, telecare, e-health, telehealth, telebehavioural health, e-therapy, e-counseling, distance counseling, remote/telerehabilitation, mHealth, telemedicine, teleconsultation, videoconferencing, telepathology, less developed country(ies), least economically developed country(ies), nonindustrialized nation(s), lower middle-income country(ies), global south, developing country(ies), underdeveloped nation (s), and third-world country(ies)/nation(s). The searches were performed on 16 February, 2017. An example of one search string used is available in Supplementary File 1.

### 2.3. Search Results

A total of 267 electronic articles were retrieved from the databases searched (Web of Science N=48; Google Scholar N=156; Psych INFO (Ovid) N=7; PubMed N=11; Springer Link N=45). Also, the hand search yielded 8 additional relevant articles on the topic under investigation. This resulted in a sum total of 275 retrieved research articles. Of the 275 initially retrieved articles, six were duplicates and a further 240 were excluded on the basis of their titles and/or abstracts,* as *they did not fulfil the inclusion criteria specified in Section 2.1.

**Figure 1:**
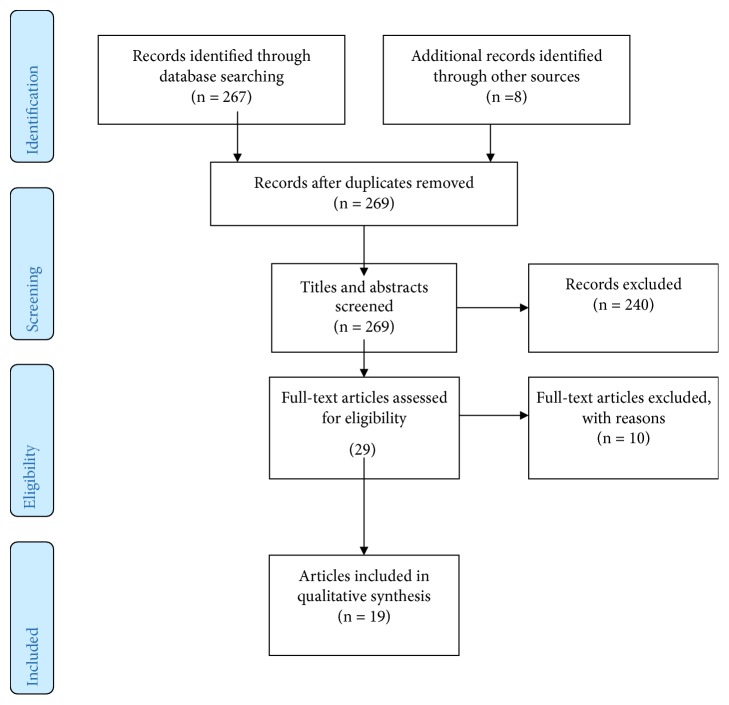
Flow-diagram of search process.

29 articles were read in full, and a further 10 were subsequently excluded. A total of 19 articles were included in the review (see Figure 1 for details of the search process and Table 1 for the included articles).

### 2.4. Extraction of Data and Synthesis of Findings

The studies included were then placed under several categories including their geographical distribution (country (s)), their main topics (illness/treatment) discussed, the study methods/designs used, and the type of e-health technology used. We also specifically examined the outcomes of the studies, looking at clinical intervention studies/randomized controlled trials, project evaluation studies, surveys, and other studies separately.

## 3. Results

### 3.1. Geographical Distribution of Studies

Of the 19 included studies, eight were conducted on the Asian continent (India [2, 28–32], Syria [33], Iran [34]), three in South America (Brazil [35, 36], Peru [37]), four in Africa (South Africa [23, 38], Nigeria [39], Somaliland [40]), and one in Europe (Russia [41]). However, three of the studies were cross-continental collaborative studies conducted between several developed and developing countries [42–44].

### 3.2. Main Topic (Illness and/or Treatment) Studied

Several mental health-related issues were discussed in the 19 included articles. More than half of these studies focused on psychiatric illness and treatment in general [2, 23, 28–30, 32, 33, 38, 40, 43, 44]. Depression [34, 36] was the second most frequently discussed psychiatric topic. Other mental health challenges or treatments that were addressed included Alzheimer's dementia [35], aggression, self-injury, stereotyped behaviour [37], obsessive-compulsive disorder [41], occupational therapy [42], psychiatric-related physical relaxation therapy [31], and education-related mental health needs [39].

### 3.3. Methods/Designs Used

Eight of the studies were conducted as clinical interventions/randomized controlled trials (RCTs). The RCT is common method in medical studies, typically used to compare the outcome of two or more interventions. One central element of the method is a random allocation of patients to different interventions, which might help the researchers in identifying the actual effects of the interventions [28–31, 34–37]. Five were identified as project evaluation studies [2, 32, 33, 40, 44]. In addition, four were classified as survey studies [23, 39, 41, 43]. Furthermore, one case study [42] and one action research study [38] were found among the 19 included articles.

### 3.4. Type of E-health Technology Used

Six of the included studies examined the use of videoconferencing in conducting telemental healthcare delivery [2, 23, 32, 36, 38, 40]. Three of the studies involved the conduction of telemental healthcare with the aid of online decision support systems [28–30] and one involved the use of both SMS text messaging and bibliotherapy [34]. One study investigated the use of videoconferencing combined with e-chatting [44]. Furthermore, two studies examined online therapy [39, 41] while others studied e-counseling [43], store-and-forward technology [33], telephone follow-up calling [37], and the use of online discussion groups/forums [42], and one used both audiovisual therapy and bibliotherapy [31]. One of the studies [35] examined computerized occupational therapy as a possible telemental care delivery medium.

### 3.5. Outcomes of Studies

#### 3.5.1. Clinical Intervention/RCTs

Eight of the 19 studies were identified as clinical intervention studies/RCTs [28–31, 34–37]. Out of these eight intervention studies, five investigated the direct effect of various telemental technologies on client symptoms improvements [31, 34–37], whereas the remaining three studies examined the use of telemental decision support systems in clinical psychiatric assessment and diagnosis [28–30]. The former class of studies showed that telemental technological modalities (computerized occupational therapy, videoconferencing, SMS text messaging, audiovisual aids, telephone follow-up calling) generally were successful in contributing to improvements in symptoms of clients suffering from Alzheimer's dementia [35], depression [34, 36], obsessive-compulsive disorder [40], aggression [37], and psychiatric-related physical conditions [31], although the findings were not consistently in favour of telemental technologies (see below). The latter category of studies [28–30] further suggested that telemental decision support technologies were viable complementary tools that with some exceptions (see below) improved clinical psychiatric assessment and diagnosis. With certain exceptions, these studies suggested a high level of clinician satisfaction with the level of accuracy, time efficiency of assessment, and level of diagnostic concordance of these telemental decision support systems compared to traditional forms of assessment [28–30].

Despite the above noted positive postintervention outcomes, not all the telemental interventions reported only positive outcomes, and there were central limitations to several of the studies. For instance, one study involving an Alzheimer's dementia patient noted some lacking tests [35]. Another study reported that based on the kappa values, sensitivity, and proportions of discordant diagnoses recorded, the decision diagnostic tool being tested performed relatively poorly on some common mental disorders including depression, dysthymia, anxiety, and stress-related disorders [30]. The relatively poor performance of the diagnostic tool was observed in its low sensitivity for certain mental disorders including organic brain disorders (60%) and sexual dysfunctions (50%), and its low specificity for broad categories of disorders (48%) and neurotic and stress-related disorders (28%) [30].

In a similar study [28], poor screening sensitivity values were also recorded for child emotional disorders, depression, and autism, as well as relatively low diagnostic sensitivity values for childhood emotional disorders (36%) and autism (50%). The tool also failed to produce high positive predictive values for oppositional defiant disorder (0.33) and autism (0.5), with diagnostic disagreement between the tool and clinicians on the diagnosis of psychosis [28]. A separate study [29] also reported similar findings on a net-based diagnostic intervention tool which showed relatively low diagnostic agreement (Kappa <0.4) for most broad category disorders including neurotic and stress-related illnesses. The tool also recorded low sensitivity on stress-related disorders (0.13), anxiety disorders (0.42), GAD (0.11), panic disorders (0.44), phobic disorders (0.22), and somatoform disorders (0.42) [29]. The net-based diagnostic tool performed relatively poorly on interrater reliability on substance dependence, psychosis, mood disorders, anxiety disorders, OCD, somatoform disorders, and dissociative disorders [29].

A videoconferencing intervention study [36] further showed that although a videoconferencing group had far superior improvements in depressive symptoms, the face-to-face patient group recorded higher treatment adherence than the videoconferencing group (*X*^2^_1_ = 2.864, *p* = 0.07).

#### 3.5.2. Project Evaluation Studies

Five of the 19 included studies were evaluations of already implemented telemental healthcare projects [2, 32, 33, 40, 44]. Therefore, these studies mainly reported on various sociotechnical outcomes of the implemented telemental projects including context factors [2, 32, 44], user and institutional technology acceptance issues [2, 32, 40], suitable technical solutions and challenges [32, 33, 40, 44], and facilitative implementation factors [2, 32, 33, 44]. All these studies indicated that low-cost, readily available, culturally sensitive, and easy-to-operate mobile technologies had successfully achieved a high user acceptance and increased adoption rates in developing countries [32, 33, 40]. Furthermore, these studies have also revealed that context-sensitive telemental projects that build local capacity, collaborate with local healthcare stakeholders and organizations, and adopt all-inclusive, user-centered implementation approaches were easily implemented, sustained, and highly utilized in developing countries [2, 32, 33, 40, 44].

#### 3.5.3. Surveys

Another group of studies found within the nineteen included articles were surveys [23, 39, 41, 43]. Although these studies showed that there is a high need for telemental services (online counseling, e-mail services, videoconferencing, e-chat, online therapy) [39], they also found that a majority of clinicians in developing countries are less technically prepared to deliver telemental health services as part of their clinical routine activities [23, 43]. The studies suggested that telemental services have not yet been incorporated into official daily clinical healthcare delivery practice, and therefore clinicians involved in these services tend to conduct telemental services mostly on a part-time basis [43].

#### 3.5.4. Other Studies

In a case study [42], it was pointed out that there is a high need for collaboration among mental health clinicians working in isolated locations/specialized fields and that they successfully utilized online discussion groups to network with colleagues (27.3%), give and receive advice (40.5%), and request and share clinical material resources (19.4%). One study [38] particularly noted that there is a need to consider context-related needs during the implementation of telemental healthcare services in developing countries including community awareness, understanding clinical needs of patients and clinicians, gaining user acceptance, training staff, ensuring an adequate Internet bandwidth, and providing local technical support onsite [38]. In general, these studies found a high need and relevance for telemental services in developing countries, as well as a feasibility for implementing low-cost, locally suitable, and readily accessible, context-sensitive telemental technologies in developing countries [38, 42].

## 4. Discussion

Despite searching several major databases, we were able to find only 19 articles that fulfilled the inclusion criteria of the review (primary studies with human subjects on telemental health, from LMIC, in English). This suggests that this research topic is still in its early stages in developing countries. The heterogeneous nature of the studies in terms of topics addressed, methods used, and outcome measures makes it difficult to compare the findings of the different studies. We are surprised to see such a high proportion of studies with strong designs and that eight of the 19 studies were interventional. This finding stands somewhat in contrast to the relatively small total number of studies, as one might expect few strong studies with strong designs in an immature field. India was the country with the highest number of included articles (6), which may reflect the importance placed in India on education and technological development, but also of course the immense size of the Indian population and the need to develop new modes of serving the health needs of the country. It seems natural that most of the articles focused on general mental health issues and generalized services, as these are likely to be the most prevalent in the healthcare sectors. However, it may also indicate a rising trend of increasing prioritization of a public health approach to mental healthcare delivery within the developing world.

Videoconferencing was the most frequently studied type of technology. Altogether seven of the studies used videoconferencing as the sole technology or in combination with other technologies. This finding is expected as videoconferencing is one of the oldest and most used technologies in telemental health provision [9, 25]. Videoconferencing is today used routinely in many developed countries as a way of providing consultations with psychologists and other mental health workers at a distance [7, 17]. However, we were surprised to find that despite the seemingly advanced and expensive technical requirements to conduct videoconferencing services (e.g., good Internet connection, adequate bandwidth, specific expensive high-end technologies), this technical modality was the most commonly described telemental modality in the literature from the resource poor settings. Other types of technologies represented less frequently were online decision support systems used for instance to help diagnose mental disorders, and a few studies drew on the use of short message service (SMS), e-chatting, and telephone follow-up. In other areas of e-health in the developing world, cell phone based services appear to be strongly on the rise, in part because large parts of the population in the LMIC have access to cell phones [45, 46]. While mHealth services are becoming more available, uptake in the health services is slow and there are few quality studies comparing their use to in-person treatment or videoconferencing services [10–12].

With respect to the outcome of the studies, most of the interventional studies reported positive outcomes in terms of reduction in patients' symptoms relating to various problems, including Alzheimer's dementia, depression, and aggression. The studies describing tests of the decision support systems also described positive outcomes in terms of improved assessments and satisfaction of users. However, some of the studies [28–30, 35, 36] reported some poor postintervention outcomes, especially in the area of computerized therapy [35], decision support systems [28–30], and videoconferencing [36]. This seems to indicate a need for further testing of several alternative technical telemental modalities and a more in-depth refinement of the current technical tools being used in this area within the LMIC. The surveys that were reported showed that while there is a high need for telemental health provision, such services are often not routinely offered, partly because of a lack of available technical equipment and other resource-related issues.

This review has some important limitations. While we searched several of the major relevant databases, including PubMed and PsycINFO, and also performed a hand search, there might have been some articles that fit the inclusion criteria but that were missed. This might especially be the case since some journals published in developing countries might not be indexed by the major databases. While we included a range of highly relevant search terms in comprehensive search-strings, we did not include some search terms (i.e., the spelling with a dash of ‘tele-mental' and ‘tele-behavioral', as well as the terms ‘underserved' and ‘rural') that might have resulted in additional relevant papers. Future reviews should consider including a higher number of search terms and also more variations in spellings of these terms. Moreover, it appears that a majority of the studies, especially the interventional studies, involved the use of Western-based standardized tests and technical tools which were not particularly modified to suit the local contexts of the LMIC settings. As a consequence, there is a risk that the findings of these studies may not have comprehensively captured a complete representation of the local telemental situation and future studies should consider this point in their research designs. Most of the intervention studies included in this review were also mainly one-time studies with no replications and often utilized smaller sample sizes, thus making it difficult to ascertain the validity of their findings. We believe that, once more literature is produced on this topic in the LMIC, future reviews may be able to obtain a much larger evidence sample which may help confirm the findings of the current studies included in this review.

## 5. Conclusions

There is a rising trend in telemental activity in the LMIC. We were able to identify 19 studies that fit the inclusion criteria. While many of the included studies had a strong study design, the total number of studies with an interventional design remains small considering the large need for telemental health services and accompanying studies in the LMIC. There is clearly a need to expand such services in the LMIC, as this is one important way of reaching out to more people with high quality mental health services. There is also a need for future studies to compare telemental health studies in the LMIC with studies from the non-LMIC.

## Figures and Tables

**Table 1:** Overview of included articles.

Reference, Main author, Year, Country	Type of eHealth	Main topic, Health problem	Method, design, N	Key findings
[2] Thara R. (2012) India	Mobile teleconsultation & videoconferencing	General mental health issues and care delivery	Evaluation study	Collaborate with local NGOs and self-help groups in delivering telepsychiatry services. Educate caregivers involved in patient care and create community awareness via local key persons.

[23] Chipps J. (2012) South Africa	Videoconferencing	General mental health issues and care delivery	Survey N=69 (11 district managers, 50 hospital managers, 8 psychiatric hospital managers)	District and hospital managers were ill prepared for e-health projects in terms of policy, technical, and planning readiness. Both district and hospital mangers were well prepared for e-health projects in areas of gender and societal readiness.

[28] Malhotra S. (2015) India	Net-based psychiatric diagnostic tool, decision support systems	General mental health issues and care delivery	Clinical intervention study N = 50 children and adolescents	The decision support system showed a satisfactory diagnosis accuracy with high levels of sensitivity and specificity for most disorders assessed. There was no significant difference between the number of correct case diagnoses made by human physicians and the decision support system.

[29] Malhotra S. (2015) India	Net-based psychiatric diagnostic tool, decision support systems	General mental health issues and care delivery	Clinical intervention study N=274 adult patients	On both the screening and criteria sub-modules, the application showed acceptable diagnostic accuracy in sensitive, specificity, and negative and positive predictive values levels for a majority of mental disorders assessed. Inter-rater reliability of the application compared to other standard scales (i.e. CGI, GAF) was also high for most diagnosis, symptom severity, and patient functional status assessed.

[30] Malhotra S. (2014) India	Clinical decision support and diagnostic system	General mental health issues and care delivery	Clinical intervention study N=100	Both the screening and diagnostic sub modules showed acceptable and appropriate levels of sensitivity, specificity and positive and negative predictive values for most mental disorders assessed. User satisfaction was reported high in terms of the tool's use feasibility and shortening consultation duration.

[31] Malhotra S. (2013) India	Audiovisual aids and printed instructions	Psychiatric-related-physical therapy (self-guided relaxation exercises)	Clinical intervention study N=45 (37 patients, 8 therapists)	Patients reported increased positive outcomes after the clinical intervention as indicated in a significant reduction in HMA-A and VAS scores during follow-up. Therapist, relatives and patients reported high satisfaction with the ease of learning and skill retention after using the audio video aids and manual in a single session.

[32] Thara R. (2008) India	Mobile teleconsultation & videoconferencing	General mental health issues and care delivery	Evaluation Study	Essential factors in establishing telepsychiatry services include identifying a suitable technology, location, collaborating with local organizations, training local staff, proper case documentation and accountability, and community service awareness creation.

[33] Jefee-Bahloul H. (2015) Syria	Store-and-forward technology	General mental health issues and care delivery	Evaluation study	Store-and-forward systems are effective and sustainable collaborative tools for global mental health networking and participation among professionals and researchers. Local clinics with limited resources can leverage store-and-forward technologies to expand their services and expertise through remote partnerships with NGOs and universities in the global community.

[34] Khadivi R. (2016) Iran	Bibliotherapy, SMS	Depression	Randomized controlled trial/intervention study N=198 patients	The control group showed a significant reduction in intensity of depressive symptoms compared to the control group (p=0.01). The text messaging group showed sustained low depressive symptoms (p<0.001) compared to the booklet (p=0.003) and control (p=0.001) groups months after the study.

[35] Assis LD. (2010) Brazil	Computerized occupational therapy	Alzheimer's dementia Occupational therapy -cognitive rehabilitation	Clinical intervention study N=8 (1 dementia patient & 7 mental health specialists)	Elderly male dementia patients showed high post-intervention improvements in cognitive function and daily activities. Qualitative improvements occurred in patients' self-initiative, agility, and physical aggression.

[36] Hungerbuehle I. (2016) Brazil	Videoconferencing	Depression	Randomized controlled trial/intervention study N=107 patients	Videoconferencing is a feasible care delivery modality in clinically unsupervised settings for depressed outpatients. Severity of depression decreased significantly in both the videoconferencing (F_2_=26.57, *p*<.001) and FTF (F_2_=29.99, *p*<.001) groups. Videoconferencing showed improvements in patient outcomes, therapeutic relationship satisfaction, low defaulting, and medication compliance. However, treatment adherence was comparatively low (X^2^_1_ =2.864, *p*=.07).

[37] Oyama-Ganiko R. (2013) Peru	Telephone follow-up calling, teleconsultation	Aggression, self-injury, stereotyped behaviour among infants at risk of developmental disorders	Clinical intervention study N=180 parents with infants at risk of developmental disabilities	Combining parent training workshops with telephone follow-up calls improved patient behavioural problems and increased caregiver satisfaction with the project. Full inclusion of parents, siblings, and other household members was a success factor in the training, consumer satisfaction, and knowledge and skills retention among participants.

[38] Chipps J. (2012) South Africa	Tele-education, video conferencing	General mental health issues and care delivery	Action research study N=9 (4 psychiatrists, 5 medical officers	Videoconference education with a flexible format, administration and technical support improvement was beneficial to health staff with low psychiatry qualification/training. A bandwidth of 128 Kilobytes per second (Kbps) is required for tele-education while 384Kbps is more reliable for tele-consultation. Keys to developing new telepsychiatry services include awareness creation, practitioner acceptance, understanding physician/patient clinical needs, and onsite technical support and local coordination service provision.

[39] Adebowale OF. (2011) Nigeria	Online counseling/therapy	Education-related mental health needs	Survey N=261 (172 college students, 89 staff)	Students reported a need for online services in areas of career planning, academic/education (i.e. problem solving and effective study skills, and academic performance) and time management. Online counseling was the most preferred format (58.8%), and e-mail communication (asynchronous) was the modality selected by a majority of students (57.1%). Most students had a positive perception toward introducing a university online guidance and counseling service (89.5%).

[40] Abdi Y. (2011) Somaliland	Skype-based telepsychiatry, Internet, videoconferencing	General mental health issues and care delivery	Evaluation study N=132	A Skype-based telepsychiatry service was found to be a cheap and effective substitute for mental health service delivery in developing countries. Clinics indicated the service has improved their credibility and increase participant satisfaction and the number patients attending these clinics.

[41] Moritz S. (2013) Russia	Online therapy	Obsessive-compulsive disorder (OCD)	Survey study N=72 OCD patients	Online Association Splitting (CBT intervention) is found to be feasible and effective in Russian speaking subjects. AS group is superior to a waitlist control condition for the improvement of obsession and depression.

[42] Dieleman C. (2013) Cross-continental study	Online discussion groups	Occupational therapy	Case study N=2494 group members	Isolated locations of practice were a push factor in health professionals' keenness to connect with colleagues via online forums. The most active participants in the online forum were from the United Kingdom (88.2%, n=1485). Seeking and giving advice (40.5%), networking (27.3%), requesting and sharing material resources (19.4%) were the most recorded activities on the online forum.

[43] Finn J. (2010) Cross-continental study	E-counseling	General mental health issues and care delivery	Survey N=93 E-counselors (80 males, 7 females)	Most E-counselors (74%) reported more satisfaction with the use of email (87%) and chat messaging (88%) and believed it was equally effective (55%) as face-to-face (36%) consultations. A majority of e-counselors reported no formal training (94%) or supervision (56.5%) in their online practice as most conducted this as a part-time supplement to their regular face-to-face consultations.

[44] Savin DM. (2013) Cross-continental study	Teleconsultation & training (Skype, e-mails, e-chatting, videoconferencing)	General mental health issues and care delivery	Evaluation study N=35 (29 Cambodian psychiatry residents, 2 faculty, 4 American residents)	A long-term consultant-consultee relationships based on strong commitment to initiate and sustain a successful collaboration is an essential interdisciplinary telepsychiatry implementation requirement. Cultural demographics shape social interactions between consultants and consultees and the solutions formulated from such interactions.

## Supplementary Materials

Supplementary Materials
** Supplementary File 1. Search string used for PubMed search. PubMed. **Date Run: 16/02/2017. (Telepsychiatry[All Fields] OR “Telemental Health”[All Fields] OR “E-Mental Health”[All Fields] OR (Behavioural[All Fields] AND (“telemedicine”[MeSH Terms] OR “telemedicine”[All Fields])) OR (Telebehavioural[All Fields] AND (“health”[MeSH Terms] OR “health”[All Fields])) OR “Online Therapy”[All Fields] OR “Computer Assisted Therapy”[All Fields]) AND (“Developing Countries”[All Fields] OR “Lower middle income countries”[All Fields] OR “Third World Nations”[All Fields] OR “Developing World”[All Fields]) AND (“2000/01/01”[PDAT]: “2017/12/31”[PDAT]). Limits: English language, species: humans, publication dates: 2000-2017.

## References

[1] Collins P. Y., Patel V., Joestl S. S. (2011). Grand challenges in global mental health. *Nature*.

[2] Thara R. (2012). Using mobile telepsychiatry to close the mental health gap. *Current Psychiatry Reports*.

[3] Semrau M., Evans-Lacko S., Alem A. (2015). Strengthening mental health systems in low- and middle-income countries: The Emerald programme. *BMC Medicine*.

[4] World Bank Data Team New Country Classifications by Income Level: 2017-2018. https://blogs.worldbank.org/opendata/new-country-classifications-income-level-2017-2018.

[5] Oyeyemi S. O., Wynn R. (2015). The use of cell phones and radio communication systems to reduce delays in getting help for pregnant women in low- and middle-income countries: a scoping review. *Global Health Action*.

[6] Wynn R., Kwabia E., Osei-Bonsu F. (2016). Internet-based provider-patient communication in Ghana: recent findings. *International Journal of Integrated Care*.

[7] Lauckner C., Whitten P. (2016). The state and sustainability of telepsychiatric programs. *The Journal of Behavioral Health Services & Research*.

[8] The American Psychiatric Association and American Telemedicine Association Best practices in videoconferencing-based telemental health. https://www.psychiatry.org/psychiatrists/practice/telepsychiatry/blog/apa-and-ata-release-new-telemental-health-guide.

[9] Yellowlees P., Richard Chan S., Burke Parish M. (2015). The hybrid doctor–patient relationship in the age of technology – Telepsychiatry consultations and the use of virtual space. *International Review of Psychiatry*.

[10] Hilty D. M., Chan S., Hwang T., Wong A., Bauer A. M. (2017). Advances in mobile mental health: opportunities and implications for the spectrum of e-mental health services. *mHealth*.

[11] Chan S., Torous J., Hinton L., Yellowlees P. (2014). Mobile Tele-Mental Health: Increasing Applications and a Move to Hybrid Models of Care. *Healthcare*.

[12] Chan S., Godwin H., Gonzalez A., Yellowlees P. M., Hilty D. M. (2017). Review of Use and Integration of Mobile Apps Into Psychiatric Treatments. *Current Psychiatry Reports*.

[13] Langarizadeh M., Tabatabaei M. S., Tavakol K., Naghipour M., Rostami A., Moghbeli F. (2017). Telemental health care, an effective alternative to conventional mental care: A systematic review. *Acta Informatica Medica*.

[14] Hilty D. M., Ferrer D. C., Parish M. B., Johnston B., Callahan E. J., Yellowlees P. M. (2013). The effectiveness of telemental health: a 2013 review. *Telemedicine and e-Health*.

[15] Brooks E., Turvey C., Augusterfer E. F. (2013). Provider barriers to telemental health: Obstacles overcome, obstacles remaining. *Telemedicine and e-Health*.

[16] Hilty D. M., Rabinowitz T., McCarron R. M. (2018). An Update on Telepsychiatry and How It Can Leverage Collaborative, Stepped, and Integrated Services to Primary Care. *Psychosomatics*.

[17] Hubley S., Lynch S. B., Schneck C., Thomas M., Shore J. (2016). Review of key telepsychiatry outcomes. *World Journal of Psychiatry*.

[18] Hyler S. E., Gangure D. P., Batchelder S. T. (2005). Can telepsychiatry replace in-person psychiatric assessments? A review and meta-analysis of comparison studies. *CNS Spectrums*.

[19] Löhr H., Wynn R., Rosenvinge J. (2007). E-therapy as an adjunct to face-to-face therapy in the treatment of patients suffering from chronic psychiatric disorders. *Journal on Information Technology in Healthcare*.

[20] Salmoiraghi A., Hussain S. (2015). A Systematic Review of the Use of Telepsychiatry in Acute Settings. *Journal of Psychiatric Practice*.

[21] Koblauch H., Reinhardt S. M., Lissau W., Jensen P.-L. (2018). The effect of telepsychiatric modalities on reduction of readmissions in psychiatric settings: A systematic review. *Journal of Telemedicine and Telecare*.

[22] Garcia-Lizana F., Munoz-Mayorga I. (2010). What about telepsychiatry? A systematic review. *“Primary Care Companion to The Journal of Clinical Psychiatry”*.

[23] Chipps J., Mars M. (2012). Readiness of health-care institutions in KwaZulu-Natal to implement telepsychiatry. *Journal of Telemedicine and Telecare*.

[24] Shore J. H. (2013). Telepsychiatry: Videoconferencing in the delivery of psychiatric care. *The American Journal of Psychiatry*.

[25] Wynn R., Bergvik S., Pettersen G., Fossum S. (2012). Clinicians’ experiences with videoconferencing in psychiatry. *Studies in Health Technology and Informatics*.

[26] Wynn R., Hagen K., Friborg O. (2012). Videoconferencing at a centre for rare disorders: User satisfaction and user participation. *Acta Paediatrica*.

[27] Liberati A., Altman D. G., Tetzlaff J. (2009). The PRISMA statement for reporting systematic reviews and meta-analyses of studies that evaluate health care interventions: explanation and elaboration. *PLoS Medicine*.

[28] Malhotra S., Chakrabarti S., Shah R., Mehta A., Gupta A., Sharma M. (2015). A novel screening and diagnostic tool for child and adolescent psychiatric disorders for telepsychiatry. *Indian Journal of Psychological Medicine*.

[29] Malhotra S., Chakrabarti S., Shah R., Sharma M., Sharma K., Singh H. (2015). Diagnostic accuracy and feasibility of a net-based application for diagnosing common psychiatric disorders. *Psychiatry Research*.

[30] Malhotra S., Chakrabarti S., Shah R. (2014). Development of a novel diagnostic system for a telepsychiatric application: a pilot validation study. *BMC Research Notes*.

[31] Malhotra S., Chakrabarti S., Gupta A. (2013). A self-guided relaxation module for telepsychiatric services: Development, usefulness, and feasibility. *International Journal of Psychiatry in Medicine*.

[32] Thara R., John S., Rao K. (2008). Telepsychiatry in Chennai, India: The SCARF experience. *Behavioral Sciences & the Law*.

[33] Jefee-Bahloul H., Barkil-Oteo A., Shukair N., Alraas W., Mahasneh W. (2016). Using a store-and-forward system to provide global telemental health supervision and training: A case from Syria. *Academic Psychiatry*.

[34] Taleban R., Zamani A., Moafi M., Jiryaee N., Khadivi R. (2016). Applications of text messaging, and bibliotherapy for treatment of patients affected by depressive symptoms. *International Journal of Preventive Medicine*.

[35] De Oliveira Assis L., Tirado M. G. A., De Melo Pertence A. E., Pereira L. S. M., Mancini M. C. (2010). Evaluation of cognitive technologies in geriatric rehabilitation: A case study pilot project. *Occupational Therapy International*.

[36] Hungerbuehler I., Valiengo L., Loch A. A., Rössler W., Gattaz W. F. (2016). Home-Based Psychiatric Outpatient Care Through Videoconferencing for Depression: A Randomized Controlled Follow-Up Trial. *JMIR Mental Health*.

[37] Oyama-Ganiko R., Mayo-Ortega L., Schroeder S., LeBlanc J. (2013). Early distance intervention and follow-up for families of infants and toddlers at risk for developmental disabilities and severe behavior problems in Peru. *Revista Educação Especial*.

[38] Chipps J., Ramlall S., Madigoe T., King H., Mars M. (2012). Developing telepsychiatry services in KwaZulu-Natal – an action research study. *African Journal of Psychiatry*.

[39] Adebowale O. F., Popoola B. I. (2011). Prospects and Challenges of Online Guidance and Counselling Services in a Nigerian University. *International Journal for the Advancement of Counselling*.

[40] Abdi Y. A., Elmi J. Y. (2011). Internet based telepsychiatry: A pilot case in Somaliland. *Medicine, Conflict and Survival*.

[41] Moritz S., Russu R. (2013). Further evidence for the efficacy of association splitting in obsessive-compulsive disorder. An internet study in a Russian-speaking sample. *Journal of Obsessive-Compulsive and Related Disorders*.

[42] Dieleman C., Duncan E. A. (2013). Investigating the purpose of an online discussion group for health professionals: A case example from forensic occupational therapy. *BMC Health Services Research*.

[43] Finn J., Barak A. (2010). A descriptive study of e-counsellor attitudes, ethics, and practice. *Counselling and Psychotherapy Research*.

[44] Savin D. M., Legha R. K., Cordaro A. R. (2013). Spanning distance and culture in psychiatric education: A teleconferencing collaboration between cambodia and the United States. *Academic Psychiatry*.

[45] Oyeyemi S. O., Wynn R. (2014). Giving cell phones to pregnant women and improving services may increase primary health facility utilization: A case-control study of a Nigerian project. *Reproductive Health*.

[46] International Telecommunications Union (ITU) ICT facts and figures 2017. https://www.itu.int/en/ITU-D/Statistics/Documents/facts/ICTFactsFigures2017.pdf.

