# Supplementary material for: Telemental Health in Low- and Middle-Income Countries: A Systematic Review
Source: Int J Telemed Appl. 2018 Nov 1;2018:9602821. doi: 10.1155/2018/9602821 (PMC6241375; doi:10.1155/2018/9602821)
Supplement: Supplementary Materials — Supplementary File 1. Search string used for PubMed search. PubMed. Date Run: 16/02/2017. (Telepsychiatry[All Fields] OR “Telemental Health”[All Fields] OR “E-Mental Health”[All Fields] OR (Behavioural[All Fields] AND (“telemedicine”[MeSH Terms] OR “telemedicine”[All Fields])) OR (Telebehavioural[All Fields] AND (“health”[MeSH Terms] OR “health”[All Fields])) OR “Online Therapy”[All Fields] OR “Computer Assisted Therapy”[All Fields]) AND (“Developing Countries”[All Fields] OR “Lower middle income countries”[All Fields] OR “Third World Nations”[All Fields] OR “Developing World”[All Fields]) AND (“2000/01/01”[PDAT]: “2017/12/31”[PDAT]). Limits: English language, species: humans, publication dates: 2000-2017. [file 9602821.f1.docx]

**Supplementary file 1. Search string used for PubMed search**

**Pubmed**

Date Run: 16/02/2017

(Telepsychiatry[All Fields] OR "Telemental Health"[All Fields] OR "E-Mental Health"[All

Fields] OR (Behavioural[All Fields] AND ("telemedicine"[MeSH Terms] OR "telemedicine"[All

Fields])) OR (Telebehavioural[All Fields] AND ("health"[MeSH Terms] OR "health"[All

Fields])) OR "Online Therapy"[All Fields] OR "Computer Assisted Therapy"[All Fields]) AND

("Developing Countries"[All Fields] OR "Lower middle income countries"[All Fields] OR "Third

World Nations"[All Fields] OR "Developing World"[All Fields]) AND ("2000/01/01"[PDAT] :

"2017/12/31"[PDAT])

Limits: English Language, Species: Humans, Publication Dates: 2000-2017
